# Perioperative and Long‐Term Functional Outcomes of Children With Early Diagnosis Versus Late Diagnosis of Hirschsprung′s Disease: A 30‐Year Monocentric Retrospective Study

**DOI:** 10.1155/ijpe/5593406

**Published:** 2026-06-12

**Authors:** Kamal El Haissoufi, Francesca Nascimben, Anne Lehn, Isabelle Chevalier, Raphael Moog, Rossella Angotti, Francesco Molinaro, François Becmeur, Isabelle Talon

**Affiliations:** ^1^ Department of Pediatric Surgery, University Hospital of Strasbourg, Strasbourg, France, chru-strasbourg.fr; ^2^ Faculty of Medicine and Pharmacy, Mohammed 1st University, Oujda, Morocco, ump.ma; ^3^ Division of Pediatric Surgery, Department of Medical, Surgical and Neurological Sciences, University of Siena, Siena, Italy, unisi.it

**Keywords:** children, early diagnosis, functional outcomes, Hirschsprung′s disease, late diagnosis

## Abstract

**Purpose:**

The purpose of this study was to compare the perioperative course and long‐term functional outcomes of children with early and late diagnosis of Hirschsprung′s disease (HD).

**Methods:**

This retrospective study included patients affected by HD who underwent pull‐through between 1984 and 2015. Patients were classified into two groups: early diagnosis of HD (EDHD) before 1 year of age and late diagnosis of HD (LDHD) after 1 year. Functional outcomes at 3, 6, and 9 years after surgery were analyzed.

**Results:**

One hundred and five patients, 83.8% with EDHD and 16.2% with LDHD. Abdominal distension and vomiting were the main presenting symptoms of EDHD, whereas chronic constipation was the primary symptom of LDHD. Preoperative intestinal obstruction occurred in 59.1% of EDHD versus 5.9% of LDHD (*p* < 0.0001). Diverting colostomy was performed in 31.8% of EDHD versus 5.9% in LDHD (*p* = 0.03). Anal strictures needing anal dilatation occurred in 30.7% of EDHD versus 5.9% in LDHD (*p* = 0.03). At 3 years of follow‐up, there was a statistical difference in terms of constipation (10% in EDHD vs. 23.5% in LDHD; *p* = 0.04), but not in terms of soiling (30.7% vs. 29.4%; *p* = 0.65); 9 years after surgery, comparable results were shown in terms of both constipation and soiling.

**Conclusion:**

Long‐term functional outcome is generally comparable between patients with EDHD and LDHD.

**Summary**

• This is the first study comparing the long‐term functional outcome between patients with an early and late diagnosis of HD.
• It has been found that even if the immediate and short‐term outcomes after a neonatal surgery are better than those after a late treatment, there are no significant differences in terms of quality of life.
• We highlight the importance of early treatment, but also of a strict long‐term follow‐up to ensure the best outcome possible.

## 1. Introduction

Hirschsprung′s disease (HD) is defined as a congenital condition where the ganglion cells that innervate the colon are missing in the distal bowel [1]. Its incidence is roughly about 1 in 5000 live births [2]. The disease is responsible for functional bowel obstruction which is mainly seen in the neonatal period [2]. After the diagnosis is established and the obstruction is effectively managed, children undergo surgical pull‐through (PT) which is usually performed by resecting the aganglionic bowel segment [2, 3]. Long‐term bowel function after definitive surgery is still the main issue for many children affected by HD, with important consequences in their quality of life [4, 5].

Children with late diagnosis of HD (LDHD) often suffer from a longer period of subocclusion states before definitive diagnosis and surgical treatment, meaning a higher risk of Hirschsprung′s disease–associated enterocolitis (HAEC), especially if associated with other symptoms such as fever or abdominal distention. Because of the rarity of this condition, their long‐term functional evolution is poorly known [3]. Results of a few previous works which have investigated whether there are changes in postoperative functional course between children with early diagnosis of HD (EDHD) and those with LDHD were controversial [4]. We hypothesized that a normal colon above the pathological distal bowel that remained dilated preoperatively for a longer time and without decompressive measures may negatively impact long‐term bowel function after surgery.

Through the present work, we aim to evaluate the perioperative course of children affected by HD and to compare the long‐term functional outcomes at 3, 6, and 9 years after PT between children with EDHD and those with LDHD.

## 2. Material and Methods

### 2.1. Patients

All children with a confirmed diagnosis of HD over a 30‐year period between January 1984 and September 2015 in the Department of Pediatric Surgery of our academic institution, University Hospital of Strasbourg in France, were evaluated in this retrospective single‐centered cohort study. We included in the present study all children aged less than 16 years old at diagnosis, of both genders, surgically treated because of HD with a follow‐up period of at least 3 years after surgery. The included patients were divided into two groups based on age at diagnosis. “Age at diagnosis” refers to the age of the child being recognized as having HD or at which the child was presumptively diagnosed and managed accordingly. The first group included patients with an EDHD, while the second group included all the others with LDHD. EDHD is defined as HD diagnosed during the first year of life, while LDHD is defined as HD diagnosed at or over 1 year of age. Patients with total colonic aganglionosis, those treated surgically at other hospitals, and those with a follow‐up period shorter than 3 years were excluded from the study.

### 2.2. Demographic Data, Clinical Presentation, and Diagnosis of HD

Medical records of enrolled patients were retrospectively reviewed, and all the following data were analyzed: gender, gestational age, birth weight, age at meconium evacuation (history of delayed passage of meconium beyond 48 h of age), associated anomalies, association with Trisomy 21, family history of HD, HD type according to aganglionic colon segment, and age at diagnosis. Diagnosis of HD was confirmed by a rectal suction biopsy (RSB). In case of equivocal results, RSB could be repeated twice, or a full‐thickness biopsy (FTB) could be scheduled. Patients underwent a contrast enema to define the length of the aganglionic segment and the type of HD. Clinical presentation, especially symptoms related to intestinal obstruction or HAEC, such as abdominal distention, fever, foul‐smelling diarrhea, vomiting, bloody stool, dehydration, laboratory tests (leukocyte and C‐reactive protein), and imaging exams, was also evaluated.

### 2.3. Preoperative Complications and Management

Preoperative complications included HAEC, intestinal obstruction, blood culture–proven sepsis, and perforation. Appropriate intravenous antibiotic therapy was administered for children with HAEC and sepsis. Bowel obstruction was managed by colorectal nursing before definitive surgery. Colostomy was indicated for patients with no response to nursing, with severe enterocolitis, or if a perforation was suspected and confirmed intraoperatively based on the preferences of the surgeon. Also, definitive surgery was performed shortly after diagnosis confirmation for all patients.

### 2.4. Surgical Data and Postoperative Complications

Among different types of definitive surgery, the Soave PT or Duhamel technique with an open abdominal approach was initially performed. Recently, transanal endorectal pull‐through (TEPT) with or without laparoscopic assistance replaced the first ones. Age and weight at definitive surgery, intraoperative complications, days at oral feeding after surgery, and length of hospital stay (LOS) were analyzed. Possible postoperative complications included anastomotic leakage, anal stricture (defined as a narrowing of the anal canal causing symptoms such as difficult or painful to expel stools, narrow stool, or constipation, diagnosed via clinical history and physical examination requiring anal dilatation), eventration, enterocutaneous fistula, adhesive bowel obstruction (an emergent condition characterized by physical intestinal blockage presenting with abdominal pain, distention, vomiting, obstipation, no peristalsis, or signs of peritonitis), anal excoriation, and postoperative HAEC.

### 2.5. Outcomes and Long‐Term Follow‐Up

All children underwent regular follow‐up. Functional results were collected, and HD surgery implications on the quality of life of these patients were analyzed. Bowel function was defined using variables related to clinical aspects of the patient′s stooling patterns, namely, the presence of constipation and soiling according to the Krickenbeck International Classification. Constipation, defined as difficult, painful, infrequent, or incomplete defecation and diagnosed through a careful medical history, the classification of symptoms by the Rome IV Criteria, and a brief physical examination, was evaluated according to the response of the child to management measures (diet modification, laxative requirement, or resistance). Similarly, the severity of soiling was measured by its frequency and its impact on the social life of the patient (Table 1) [6]. Manometry was not routinely performed as a postoperative test, so it was not included in the manuscript as an outcome. Based on this, functional outcome was compared between the two groups at 3, 6, and 9 years after definitive surgery.

**Table 1:** Krickenbeck International Classification for postoperative functional outcomes (soiling and constipation) [6].

**Soiling**	
Grade 1	Occasionally (once or twice per week)
Grade 2	Every day, no social problem
Grade 3	Constant, social problem
**Constipation**	
Grade 1	Manageable by changes in diet
Grade 2	Requires laxative
Grade 3	Resistant to laxatives and diet

### 2.6. Statistical Analysis

A descriptive analysis was performed. Continuous variables were presented by averages with standard deviations, and they were compared through Student *t*‐test or nonparametric tests; qualitative variables were described as frequencies and percentages, and they were compared by using chi‐square or Fisher test. A *p* value less than 0.05 was considered statistically significant. All statistical analyses were performed using SPSS 21.0.

## 3. Results

**Figure 1:**
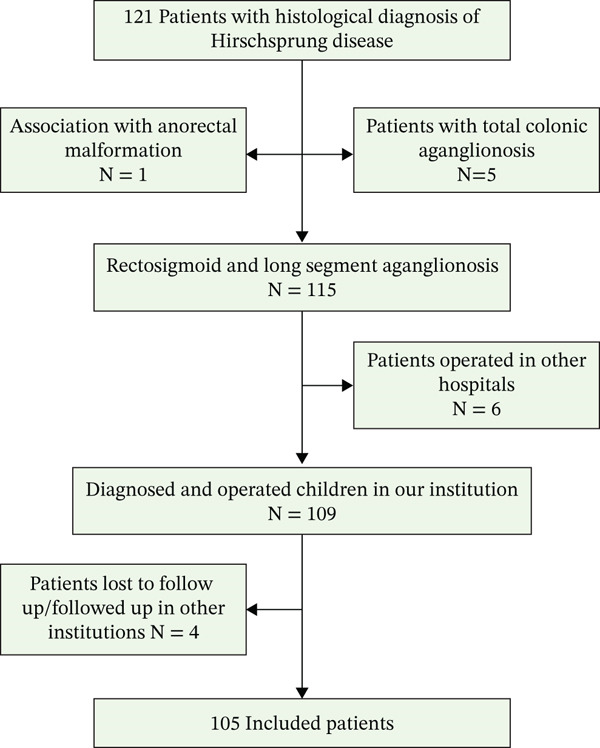
Flowchart showing the process of selecting eligible patients to be included in the study.

Overall, 118 patients with a histological diagnosis of HD were retrieved from our databases. The final evaluable population included 105 patients after 13 patient withdrawals (Figure 1). Among the remaining patients, 88 (83.8%) had HD diagnosed within the first year of age and were included in the EDHD group, whereas the other 17 (16.2%) received a diagnosis of HD after the first year of age and were included in the LDHD group.

Seventy‐eight patients (74.3%) were male, and 27 (25.7%) were female. Nine patients (8.6%) were born prematurely. Mean birth weight was 3.1 [1.4–3.9] kg. HD was sporadic in 83 cases (79%), while 22 patients (21%) had associated anomalies, mostly consisting of cardiac malformations in eight cases (7.6%), psychoneuronal disorders in eight cases (7.6%), and genital and urinary tract anomalies in five cases (4.8%). Down syndrome was associated with HD in six patients (5.7%). Also, there were 11 cases (10.5%) of familiarity for HD within our patients.

Mean age at first reported clinical signs was 29 days (1 day–2 years). Remarkably, abdominal distension was the most common sign of revelation (78.1%), followed by vomiting (59%) and constipation (49.5%). Interestingly, delayed passage of meconium beyond 48 h after birth was seen in 45 patients (42.9%). Comparatively, a significant predominance of boys was noticed within the EDHD group (*p* = 0.03). Also, abdominal distension and vomiting represent the main signs and symptoms of early diagnosed children with HD (*p* < 0.0001), whereas patients with LDHD presented more chronic constipation as more frequent sign (*p* < 0.0001). Regarding meconium evacuation, a delayed passage beyond 48 h after birth was mainly observed in the EDHD group but without any statistical significance. Demographic data and clinical presentation of both groups are presented comparatively in Table 2.

**Table 2:** Patient demographics and clinical characteristics.

Characteristics	EDHD group *N*(%)	LDHD group *N*(%)	*p*value
Sex			0.03
Male	69 (78.4)	9 (53)	
Female	19 (21.6)	8 (47)	
Gestational age			0.09
Preterm	6 (6.8)	3 (17.6)	
Term	79 (89.8)	10 (58.8)	
Missing	3 (3.4)	4 (23.5)	
Birth weight (g)	3148 ± 436	3126 ± 750	0.92
Missing	3 (3.4)	5 (29.4)	
Familiarity	11 (12.5)	0 (0)	0.13
Comorbidity	19 (21.6)	3 (17.6)	0.71
Down syndrome	4 (4.5)	2 (11.7)	0.25
Meconium evacuation			
Within 48 h after birth	30 (34.1)	5 (29.4)	0.05
Beyond 48 h after birth	44 (50)	1 (5.9)	
Missing	14 (15.9)	11 (64.7)	
Clinical presentation			
Abdominal distension	76 (86.4)	6 (35.3)	< 0.0001
Vomiting	62 (70.4)	0 (0)	< 0.0001
Constipation	36 (40.9)	16 (94.1)	< 0.0001
Age of diagnosis (days)	64 ± 84	979 ± 695	< 0.0001

*Note:* Values are presented as means ± standard deviation or as *N* (%).

Abbreviations: EDHD, early diagnosis of Hirschsprung′s disease; LDHD, late diagnosis of Hirschsprung′s disease.

All included patients underwent a contrast enema study and RSB before surgery. Notably, 35 of the first biopsies (33.3%) failed to make a diagnosis because of inadequate sampling of the specimen, and further biopsies were needed to confirm the histological diagnosis of HD. Rectosigmoid aganglionosis was the most encountered form of the disease in 76 patients (72.4%). Otherwise, long‐segment aganglionosis and short‐segment HD were seen in 21 (20%) and 8 (7.6%) patients, respectively. Additionally, the mean age of patients at diagnosis was 7 ± 14.5 months. The earliest diagnosis was made at the age of 3 days, and the oldest patient at diagnosis was 8 years old. The mean time between histological diagnosis and definitive treatment was 78 ± 123 days. During this period, the dilated colon was decompressed initially by reinforced laxative measures and daily nursing in all children. However, a temporary colostomy was mandatory in 28 children of the EDHD group (31.8%) and only in 1 patient of the LDHD group (5.9%) (*p* = 0.03). Remarkably, intestinal obstruction was more commonly encountered in patients with EDHD before performing definitive surgery (*p* < 0.0001).

Also, 17 children (16.2%) presented HAEC preoperatively with a median age of 20 days (2 days–28 months). Preoperative perforation of the colon was seen in seven children (6.7%) who all belonged to the EDHD group. The median age of perforation occurrence was 3 days (2 days–7 months). In 13 patients with diverting colostomy (44.8%), stoma complications occurred: 6 cases of pericolostomic eventration (20.7%), 2 cases of stomal prolapse (6.9%), 2 cases of adhesive intestinal obstruction (6.9%), 1 stomal stenosis (3.4%), 1 case of postoperative peritonitis (3.4%), and 1 case of abdominal wall infection (3.4%). Subsequently, 10 patients underwent redo surgery including 3 colostomy repairs (10.3%), 1 peritoneal lavage (3.4%), and 6 eventration repairs (20.7%).

After decompression of the normal colon, whether through nursing or carrying out a colostomy when mandatory, a definitive surgery consisting of resection of the aganglionic segment was performed for all children. The mean age and weight at surgery were 9.5 ± 16.3 months and 6.9 ± 3.8 kg, respectively. TEPT was the most performed in 57 children (54.3%), followed by laparoscopic‐assisted endorectal pull‐through (LAEPT) in 26 children (24.8%). There were no intraoperative complications nor bleeding incidents requiring transfusion.

After surgery, oral refeeding was restored after a mean of 2.2 ± 2.3 days.

Both perineal excoriation which was the most common complication (52.4%) and postoperative HAEC which was diagnosed in four patients (4.5%) did not show any significant difference between the two groups, with an incidence of 54.5% versus 41.2% (*p* = 0.31) and 4.5% versus 0% (*p* = 0.49), respectively. On the other hand, anal stricture with need of anal dilatation was mostly seen in patients of the EDHD group than in the LDHD group (30.7% vs. 5.9%, *p* = 0.03); interestingly, the rate of stenosis was statistically significantly higher (*p* = 0.03) in LDHD (28.9%) than in EDHD (13.3%) when using 6 months of age as threshold.

Other postoperative complications included megarectal Duhamel pouches in 13 patients (12.4%), adhesive intestinal obstruction in 6 patients (5.7%), 2 retained aganglionic segments (1.9%), 2 mucosal rectal prolapses (1.9%), anastomotic leakage in 1 patient (0.9%), and rectocutaneous fistula with abscess formation in 1 patient (0.9%). The mean LOS was 14.8 ± 12.5 days, 15.7 ± 13 days in the EDHD group and 9.8 ± 8.8 days in the LDHD group (*p* = 0.008). Data regarding diagnosis, preoperative evolution, treatment, and postoperative course are represented comparatively between the two groups in Table 3.

**Table 3:** Diagnosis and preoperative characteristics, treatment, and postoperative course.

	EDHD group *N*(%)	LDHD group *N*(%)	*p*value
Diagnosis			
Short‐segment HD	2 (2.3)	6 (35.3)	**< 0.001**
Rectosigmoid aganglionosis	66 (75)	10 (58.8)	**< 0.001**
Long‐segment aganglionosis	20 (22.7)	1 (5.9)	**< 0.001**
Preoperative complications			
Intestinal obstruction	52 (59.1)	1 (5.9)	**< 0.001**
HAEC	16 (18.2)	1 (5.9)	0.29
Colon perforation	7 (7.9)	0 (0)	0.59
Colostomy	28 (31.8)	1 (5.9)	**0.03**
Surgical technique			
TEPT	50 (56.8)	7 (41.1)	**< 0.05**
LAEPT	20 (22.7)	6 (35.3)	**0.004**
Duhamel	17 (19.3)	1 (5.9)	**< 0.05**
Other	1 (1.1)	3 (17.6)	**< 0.05**
Postoperative oral feeding (days)	2.2 ± 2	2.2 ± 3.2	0.68
Postoperative complications			
Perineal excoriation	48 (54.5)	7 (41.2)	0.31
HAEC	4 (4.5)	0 (0)	0.49
Anal stricture	27 (30.7)	1 (5.9)	**0.03**
Adhesive intestinal obstruction	6 (6.8)	0 (0)	0.33
Mucosal rectal prolapse	2 (2.3)	0 (0)	0.70
Transition zone pull‐through	2 (2.3)	0 (0)	0.70
Megarectal Duhamel pouch	12 (13.6)	1 (5.9)	0.33
Anastomotic leakage	1 (1.1)	0 (0)	0.83
Rectocutaneous fistula	0 (0)	1 (5.9)	0.16
LOS (days)	15.7 ± 13	9.8 ± 8.8	**0.008**

*Note:* Values are presented as means ± standard deviation or as *N* (%). Boldface data indicate *p* value < 0.05.

Abbreviations: EDHD, early diagnosis of Hirschsprung′s disease; HAEC, Hirschsprung′s disease–associated enterocolitis; HD, Hirschsprung′s disease; LAEPT, laparoscopic‐assisted endorectal pull‐through; LDHD, late diagnosis of Hirschsprung′s disease; LOS, length of hospital stay; *N*, number; TEPT, transanal endorectal pull‐through.

A univariate analysis based on the Krickenbeck International Classification for constipation and soiling between the two groups at 3, 6, and 9 years postoperatively was performed. Interestingly, soiling was comparatively seen, and no significance between patients with EDHD and LDHD was statistically observed. However, the occurrence of constipation was more likely encountered in children with LDHD at 3‐year evaluation during the postoperative course (*p* = 0.04). Three years and 6 years later, no constipation was seen within patients of LDHD; though, given the small number of patients in this group at final evaluation, these findings seem difficult to interpret properly. Functional outcomes of our patients are shown comparatively between the two groups in Table 4.

**Table 4:** Functional outcomes.

	EDHD group *N*(%)	LDHD group *N*(%)	*p*value
Soiling			
3 years	27 (30.7)	5 (29.4)	0.65
6 years	34 (38.6)	2 (11.7)	0.66
9 years	26 (29.5)	2 (11.7)	0.68
Constipation			
3 years	9 (10.2)	4 (23.5)	0.04
6 years	15 (17)	0 (0)	0.42
9 years	11 (12.5)	0 (0)	0.42

Abbreviations: EDHD, early diagnosis of Hirschsprung′s disease; HD, Hirschsprung′s disease; LDHD, late diagnosis of Hirschsprung′s disease; *N*, number.

*p* value< 0.05

## 4. Discussion

HD is a common congenital malformation of the enteric nervous system caused by the arrest of craniocaudal migration of neural crest cells during pregnancy, whose prenatal diagnosis is almost impossible [1, 7]. Even if it appears to have a complex genetic predisposition [8, 9] and different hypotheses including the role of oxidative stress have been proposed [10], the etiology is still unclear [1, 2]. Although HD typically presents in the newborn period, it can occasionally be diagnosed in older children or adults, showing different scenarios.

In this retrospective study, we sought to compare the perioperative course and particularly the long‐term functional outcomes after PT between patients timely diagnosed and those with LDHD. Our initial hypothesis suggested that children with LDHD who remained for a long time with a dilated ganglionic colon before definitive surgery may have an unfavorable impact on the functional outcome after PT. As for functional outcome, there was no significant difference between the two groups except for a greater tendency to constipation 3 years after surgery in the LDHD group. Furthermore, we noticed more preoperative intestinal obstruction, more preoperative intestinal perforation, and more need for colostomy before definitive surgery in the EDHD group than in LDHD. These data are in contrast to most published ones, where late diagnosis is generally associated with a higher colostomy rate. The first explanation for these different results is the surgical practice of performing a colostomy as a planned step before PT in EDHD until the 2010s: A diverting colostomy seemed to be the safest temporary strategy for patients affected by HD before definitive PT; starting in 2010, surgeons felt more and more confident in performing PT without a temporary colostomy. Moreover, advances in anesthesia, neonatal intensive care, and antibiotics allow surgeons to choose one‐step approaches.

Furthermore, we did not stratify the colostomy rate according to the type of HD for any group: Colostomy was probably more frequently performed in long‐segment aganglionosis, whose prevalence was higher in EDHD than in LDHD. Finally, late‐diagnosed patients in our cohort may have adapted their intestinal habits, enabling an elective, one‐stage procedure, while early diagnosed patients experienced more severe conditions; in fact, 100% of preoperative perforation occurred in the EDHD group. Interestingly, patients with EDHD seem to be more likely to present with preoperative and postoperative HAEC and a higher occurrence of postoperative anal stricture. These controversial data may be explained by different causes: First of all, we did not consider a strict definition of HAEC, but we took into account both severe HAEC presentations and mild ones; furthermore, at the beginning of this study period, HAEC might be misunderstood with a consequent delayed diagnosis and treatment, increasing its occurrence.

Even after successful surgical treatment of HD addressing the immediate anatomical problem, ensuring good long‐term functional outcomes—such as normal bowel habits and continence—remains a significant and ongoing challenge for the surgeons, but mostly for the patients and their families. Often, the surgeon knows that his role extends beyond the operating room into long‐term multidisciplinary care; however, whereas some healthcare providers initially hope to fix the problem with surgery, they ultimately realize after the operation the importance of education, reassurance, and support in optimizing the children′s function and quality of life. Concretely, the absence of soiling and constipation with a normal voluntary bowel movement was considered good judgment criteria for a good functional evolution [11–13]. Indeed, digestive disturbances are frequent, impacting the general well‐being and quality of life of children operated on for HD, regardless of the chosen surgical technique [5]. Previous studies to find out factors associated with good functional results after PT including age at surgery were carried out, but findings were inconclusive [12, 14]. Also, there is no clear consensus to define LDHD and allow a reliable comparison between the results of different studies. Recently, Tan et al. considered an LDHD if diagnosis is made at or over 1 year of age which appears to be the most valid and more resorted to [15, 16]. Accordingly, we have referred to the Krickenbeck International Classification and the age of 1 year as the diagnosis threshold timing to conduct our study.

In terms of preoperative management, temporary colostomy is not mandatory, but we would like to stress the role of routine rectal washout which is still a good habit for colonic preparation, especially in the LDHD group, to avoid caliber discrepancy of the colon before surgery [17].

According to different studies such as Tan et al.′s case series, children with LDHD are more likely to be exposed to major early postoperative complications such as anastomotic leakage [15, 17, 18]. On the other hand, Ostertag‐Hill et al. reported that patients with LDHD experience a similar risk of complication occurrence, stoma placement, or redo surgery [19]. The high stoma complication rate may be explained by the inclusion of both mild and major stoma complications, by the setting of performing stomas, both urgent and elective, and by the unstandardized postoperative stoma management at the beginning of the study period. Our results are partially in contrast with those reported in the literature; it is reasonable to say that the type and the timing of surgery, the presence of a temporary colostomy, the era in which the surgery occurred, surgical expertise, and evolving techniques all play a significant role in postoperative outcomes and significantly affect complication rates.

Comparable long‐term functional outcomes were reported by Ostertag‐Hill et al., who showed a constipation rate of 43% requiring laxatives among patients with a late diagnosis over a median follow‐up of 5.4 years [19]. Interestingly, a nationwide cohort study performed in the Netherlands concluded that no optimal timing for PT could be determined since age at surgery was not proven to be associated with the risk and severity of fecal incontinence and constipation in the long term among patients operated on because of HD [20]. Postoperative bowel function was reported to improve over time without a significant impact of age at surgery of HD after a follow‐up period of 10 years [14]. Also, Kastenberg et al. emphasize that surgery after the neonatal period seems to be safe with equivalent functional outcomes after three‐and‐a‐half years postoperatively compared to PT in the neonatal period [21]. Likewise, no more occurrence of preoperative HAEC or complications with a comparable functional outcome was noticed in another recent study in case of diagnosis delay beyond 1 year of age [16]. However, a normal meconium passage was more likely observed among these children, which is mainly consistent with our results [16].

Similarly, a recent meta‐analysis including 780 cases from three different countries revealed that children aged less than 2 months and half at the time of surgery seem to have worse functional course with higher rates of soiling, anastomotic leakage, and stricture [4]. Younger children at the time of surgery were more likely to present with postoperative constipation and HAEC as well [4]. Interestingly, Zhu et al. found that children operated before 3 months of age had lower diagnosis accuracy of RSB and poorer short‐term and midterm outcomes [22]. Nevertheless, Ullrich et al. conducted a multicentric study that enrolled a large population and concluded that late diagnosis is significantly associated with a greater need for postoperative interventions because of constipation or incontinence [3]. However, no differences regarding age at diagnosis were seen in terms of surgery revision or of short‐term postoperative complication rates [3].

Future strategies should focus on early and accurate diagnosis in both early suspected HD and late ones, educating the pediatricians in recognizing milder, chronic symptoms of HD, especially in specific risk groups of patients like children with Down syndrome or family history of HD, and promoting the use of standardized screening protocols including noninvasive diagnostic tools as much as possible, such as molecular or imaging biomarkers. Another important point is the importance of an appropriate individualized surgical planning regardless of the age at diagnosis to avoid overtreatment such as temporary colostomy or unnecessary complications. Future treatment guidelines could emphasize functional status over age at diagnosis when choosing surgical methods. Moreover, scientists could investigate nerve‐sparing techniques to reduce the risk of functional complications like soiling and incontinence. The final point to consider for future practice is the establishment of a dedicated HD follow‐up care team involving pediatric surgeons, gastroenterologists, psychologists, and continence nurses, along with a structured pathway to monitor delayed functional outcomes; for example, manometry should be routinely used to follow the postoperative course of these patients.

## 5. Conclusion

First of all, we highlight the importance of considering the diagnosis of HD in children over 1 year old with therapy‐resistant constipation. According to our results, we can confirm the variability of clinical presentation depending on the age of the child at diagnosis of HD. Also, we suggest that long‐term functional outcome is generally comparable between patients with EDHD and LDHD. However, a late diagnosis requires preoperative colonic preparation that is even more important as the delay is long, in order to optimize the caliber of the healthy colon, limit extensive resection of a colon that remains too large, and reduce the risk of postoperative complications.

### 5.1. Limitations of the Study

This work presents some limitations. Firstly, the sample of patients in the two groups is heterogeneous: It is difficult to perform a statistical analysis comparing two groups with different numbers of patients, especially in the LDHD group, due to the clinical presentation, where diagnostic delay in HD is uncommon. Secondly, the retrospective nature of the study. Therefore, further prospective cohorts with greater sample sizes are strongly required to discuss our results critically.

**Nomenclature**
HD: Hirschsprung′s disease
PT: pull‐through
EDHD: early diagnosis of Hirschsprung′s disease
LDHD: late diagnosis of Hirschsprung′s disease
RSB: rectal suction biopsy
FTB: full‐thickness biopsy

## Author Contributions

I.T. conceived the idea for this case series. F.N. and K.E.H. drafted the initial manuscript, reviewed and revised the manuscript, and conducted the review of the literature. I.T. coordinated and supervised data collection and critically reviewed the manuscript. I.T., A.L., R.M., F.B., and I.C. contributed to the diagnostic and therapeutic management of the patients. I.T. supervised the final writing of the manuscript.

## Funding

No funding was received for this manuscript.

## Disclosure

All authors contributed to the article and approved the submitted version.

## Ethics Statement

Ethics approval from the internal ethical committee of CHU Hautepierre, Strasbourg, France, was obtained. The parents of all patients included in the article were asked to sign a written consent in order to use their data for scientifical purpose. We conducted this study in compliance with the principles of the Declaration of Helsinki. Before performing this study, informed consent from all included patients or their parents was obtained.

## Conflicts of Interest

The authors declare no conflicts of interest.

## Data Availability

The dataset supporting the conclusions of this article is available in a repository, and it may be shared by the corresponding author upon reasonable request.
